# Adipose-derived stem cell-based treatment for acute liver failure

**DOI:** 10.1186/s13287-015-0040-2

**Published:** 2015-03-21

**Authors:** Guangfeng Chen, Yinpeng Jin, Xiujuan Shi, Yu Qiu, Yushan Zhang, Mingliang Cheng, Xiaojin Wang, Chengwei Chen, Yinxia Wu, Fuzhu Jiang, Li Li, Heng Zhou, Qingchun Fu, Xiaoqing Liu

**Affiliations:** Tenth People’s Hospital, Tongji University School of Medicine, 301 Yanchangzhong Road, Shanghai, 200072 P.R. China; Shanghai Liver Diseases Research Center, The Nanjing Military Command, 9585 Humin Road, Shanghai, 200235 P.R. China; Department of Infectious Diseases, Affiliated Hospital, Guiyang Medical College, 9 Beijing Road, Guiyang, 550004 P.R. China

## Abstract

**Introduction:**

Acute liver failure (ALF) is a highly lethal disease, for which effective therapeutic methods are limited. Although allogeneic liver transplantation is a viable treatment method for ALF, there is a serious shortage of liver donors. Recent studies suggest that stem cell transplantation is a more promising alternative. Hence, we investigate whether human adipose-derived stem cells (ASCs) have the therapeutic potential for ALF in this study based on the studies of rat models.

**Methods:**

Sprague Dawley rats were used to establish ALF models by D-galactosamine injection. These rats were randomly divided into a human ASC-treated group and a phosphate-buffered saline (PBS) control group. The human ASCs or PBS was transplanted through the spleen of rats. The indices of hepatic function and hepatic histology were dynamically detected, and the survival rates of rats were also counted. Double-fluorescence immunohistochemistry was employed to detect the ASC fate after transplantation. Moreover, both concentrated ASC conditional media and ASC lysates were transplanted through the femoral vain of rats to investigate the therapeutic potential for ALF.

**Results:**

The ASC transplantation group showed improved viability in comparison with the sham control. Histological and biochemical analysis suggested that liver morphology and function were improved in terms of cell proliferation and apoptosis. Although a plethora of ASCs persist in the spleen, the improvement in liver function was obvious. However, ASCs did not differentiate into hepatocytes after engrafting to livers within 3 days. In addition, both concentrated serum-free ASC conditional media and ASC lysates, characterized by high levels of hepatocyte growth factor and vascular endothelial growth factor, demonstrated obvious improvement in terms of high survival rates of ALF rats.

**Conclusion:**

Our data suggest that ASC transplantation has the potential for ALF treatment partly by the mechanism of secreting growth factors contributing to liver regeneration.

## Introduction

Acute liver failure (ALF) is defined as the extensive necrosis of hepatocytes caused by a variety of factors in a short time, and severe hepatic disorders eventually may lead to syndromes associating with functional failure [1-3]. ALF is also characterized by acute progression and high mortality, and effective treatments are still lacking. Although common supportive treatment and artificial liver are accepted for clinic use, their efficacies remain to be improved [4]. Liver transplantation shows relatively good efficacy but its application is limited by both the shortage of donor and expensive cost. Hepatocyte transplantation has also been applied to elevate the survival rate of animals with ALF induced by chemistry and surgery [5]. However, its clinical application was limited for the availability of human hepatocytes and it remains a challenge to amplify the primary hepatocytes after cryopreservation and resuscitation [6,7]. Hence, it is urgent to find alternative cell sources.

Stem cells represent a type of undifferentiated cells, which could be expanded extensively *in vitro* [8]. Bone marrow-derived mesenchymal stem cells (BMSCs) are an important source of adult stem cells. They have strong abilities of proliferation and differentiation, including differentiating to hepatocyte-like cells [9-11]. Recently, BMSC transplantation has shown therapeutic potentials for liver failure in both rats and pigs [12,13]. Adipose-derived stem cells (ASCs) are another important source of adult stem cells [14-17]. Although BMSCs and ASCs share similar properties, including cell surface markers, gene expression profile, immunosuppressive properties, and differentiation capacity, the proliferation rate of ASCs is higher than that of BMSCs [18-22]. However, extensive preclinical studies are needed to evaluate the ASC treatment potential for liver failure.

In this study, human ASCs were transplanted through the spleen to treat ALF rats. Biochemical indices of liver, including serum albumin (ALB), alanine aminotransferase (ALT), aspartic aminotransferase (AST), hepatocyte growth factor (HGF), vascular endothelial growth factor (VEGF), liver histological changes, and survival rate, were investigated to assess the efficacy of ASC treatment. The distribution of ASCs in the main organs and cell fate after transplantation were also detected. Moreover, both concentrated ASC conditional media and ASC lysates were transplanted through the femoral vain of rats to investigate the therapeutic potential for ALF. The obtained data provided important information for the potential application of ASC transplantation for ALF treatment.

## Methods

### Animals and cell resources

Specific pathogen-free Sprague Dawley (SD) rats (male, 120 to 140 g) at the age of 4 to 6 weeks were provided by SLAC Laboratory Animal Co., Ltd. (Shanghai, China) (license #SCXK (Hu) 2007–0005). The rats were bred within the Animal Unit of Tongji University. All experiments involving animals were performed in accordance with the National Institutes of Health Guide for the Care and Use of Laboratory Animals and approved by the Biological Research Ethics Committee of the Chinese Academy of Sciences. Human ASCs were prepared as previously described [23]. They were isolated from adipose tissues obtained from patients undergoing tumescent liposuction in accordance with procedures approved by the Ethics Committee at the Chinese Academy of Medical Sciences and Peking Union Medical College. All patients provided written informed consent. Briefly, adipose tissues obtained from the patients were washed three times by phosphate-buffered saline (PBS) with 1% penicillin/streptomycin and carefully minced by sterile operation scissors. The minced tissues were enzymatically dissociated for 45 minutes at 37°C by adding isometric 0.15% collagenase type I (Gibco, now part of Thermo Fisher Scientific, Waltham, MA, USA). The suspension was neutralized with isometric culture media and centrifuged at 500 *g* for 5 minutes. The cell pellet was resuspended in Dulbecco’s modified Eagle’s medium/F12 (DMEM/F12) media (Gibco) supplemented with 10% fetal bovine serum (FBS) (Gibco) or 10% KnockOut Serum Replacement (Invitrogen, now part of Thermo Fisher Scientific) or ASC serum-free media (Advcell; BioWiseTech Co., Ltd., Wilmington, DE, USA) at a density of 2 × 10^6^ cells/mL. Cells were maintained at 37°C in a humidified incubator supplemented with 5% CO_2_. Cells at passage 3 were used for the following experiments.

### Detection of adipose tissue-derived stem cell surface markers by using immunocytochemistry and flow cytometry

For immunocytochemistry, cells were seeded into a sixwell plate that was pre-coated with a cover glass. After 24 hours, cells were fixed for 30 minutes by using 4% paraformaldehyde and incubated with CD29-FITC, CD44-PE, CD90-FITC, CD105-PE, CD34-FITC, and CD133-PE anti-human antibodies (0.5 μL/well) for 30 minutes. Cells were washed by PBS for three times. The fluorescence was observed after photophobical incubation for 30 minutes. For flow cytometry detection, the ASCs (passage 3) were used to prepare single-cell suspension, and subsequently 2 μL of CD29-FITC, CD44-PE, CD90-FITC, CD105-PE, CD34-FITC, and CD133-PE antibodies were added into the suspension. After incubating for 30 minutes photophobically, ASCs were washed three times and detected by using a flow cytometry (BD FACSAria III; BD Biosciences, San Jose, CA, USA).

### Adipogenic, chondrogenic, and osteogenic differentiation

Human ASCs at a density of 5 × 10^3^ cells/cm^2^ were seeded into sixwell plates pre-coated with a cover glass. The cells were induced for 3 weeks by using adipogenic (#HUXMA-90031; Cyagen Biosciences Inc., Guangzhou, China), chondrogenic (#HUXMA-90041; Cyagen Biosciences Inc.), and osteogenic (#HUXMA-90021; Cyagen Biosciences Inc.) differentiation media, respectively. Adipocytes, chondrocytes, and osteocytes were observed by using Oil Red O staining, Alcian blue staining, or Alizarin red staining in accordance with the protocols of the manufacturer (Cyagen Biosciences Inc.). Briefly, for Oil Red O staining, cells were washed with PBS three times and fixed with 4% formaldehyde for 10 minutes. After incubation with Oil Red O reagent for 30 minutes, cells were washed three times with PBS and observed by using microscopy (IX71; Olympus, Tokyo, Japan). Similarly, for Alcian blue staining and Alizarin red staining, chondrocytes and osteocytes were fixed with 4% formaldehyde and incubated with Alcian blue reagent and Alizarin red reagent for 3 hours and 30 minutes at room temperature, respectively. Excessive staining reagents were removed by washing with PBS three times.

### Intrasplenic adipose tissue-derived stem cell transplantation

Fifty male SD rats were intraperitoneally injected with D-galactosamine (D-gal) at a dose of 1.4 g/kg. Thirty rats with ALF were randomly divided into an ASC treatment group and a PBS treatment group. The transverse incision of about 1 cm was obtained at the right subcostal of rats in a laminar flow cabinet on the next day of modeling. The spleen was exposed by using a smooth forceps, and 100 μL of ASC suspension (5 × 10^6^ cells) was injected into the spleen. The control group was injected with 100 μL of PBS. Then local hemostasis and wound suturation were performed. The body weight, activity, food intake, urine, hair color, muscle strength, and response to stimuli of rats were daily observed and recorded, and the survival rates were statistically analyzed. The tail blood of rats was obtained at different time points (1, 3, and 7 days after D-gal injection). Serum ALT, AST, and ALB levels were detected by using an automatic biochemical analyzer. The left hepatic lobes were obtained once the rats died or were sacrificed 7 days later after D-gal injection, and the paraffin-embedded sections of the livers were further obtained. The histological changes were observed at high magnification after hematoxylin-and-eosin (HE) staining. Ki67 staining and the terminal deoxynucleotidyl transferase dUTP nick end labeling (TUNEL) assay were used to detect cell proliferation and apoptosis, respectively.

### Tracing of transplanted human adipose tissue-derived stem cells

Human ASCs at passage 3 were infected with lentivirus expressing ZsGreen at 70% cell confluence [24]. The infection efficiency was observed after 48 hours of infection by using a fluorescence microscope (IX71; Olympus), and the cells were cultured successively. Twenty ALF model rats were randomly divided into the intrasplenic transplantation group and the vein transplantation group, and these rats were injected with 5 × 10^6^ ASCs via the spleen and femoral vein, respectively. The distribution and engraftation of ASCs in organs were dynamically detected by using immunohistochemistry. Human ALB and human alpha-fetoprotein in the ZsGreen-positive ASCs were detected by using double-fluorescence immunohistochemistry analysis. The antibodies specific against ZsGreen, human ALB, and human alpha-fetoprotein were purchased from Abcam Ltd. (Cambridge, UK).

### Intravenous concentrated adipose tissue-derived stem cell (ASC) conditional media/ASC lysate injection

For preparation of ASC conditional media, 10^6^ human ASCs were seeded into a T75 culture flask, and the media were replaced with 7 mL of DMEM/F12 media (without FBS) 12 hours later. The supernatant was collected after 24 hours of incubation. The ASC conditional media were concentrated 25 times by centrifugation at 8,000 *g* per minute by using ultrafiltration centrifugal tubes (MWCO:3 K; Sartorius, Goettingen, Germany). For preparation of ASC lysates, 10^7^ ASCs were collected in a 15-mL tube. The cells were frozen at −80°C and thawed at 37°C three times. Then 1 mL of DMEM/F12 media (without FBS) was added into the tube. After vortex for 1 minute, the supernatant was collected by centrifugation at 5,000 *g* for 5 minutes. The collected supernatant was filtrated by a 0.22-μm filter (Millipore, Billerica, MA, USA), and each rat was injected with 100 μL via the femoral vein. The blood of rats was harvested after 3 days of injection, and the serum was collected by centrifugation at 4°C. The levels of HGF and VEGF in the serum, concentrated ASC conditional media, and ASC lysates were detected by using an enzyme-linked immunosorbent assay (ELISA) kit (Shanghai ExCell Biology, Inc., Shanghai, China) in accordance with product instruction manuals.

### Statistical analysis

Data were represented as mean ± standard error of mean. Survival rate was analyzed by using Kaplan-Meier analysis. Data were analyzed by using Statistical Product and Service Solutions (SPSS) version 19.0 (SPSS, Inc., Chicago, IL, USA).

## Results

### Characterization of adipose tissue-derived stem cells for transplantation

**Figure 1:**
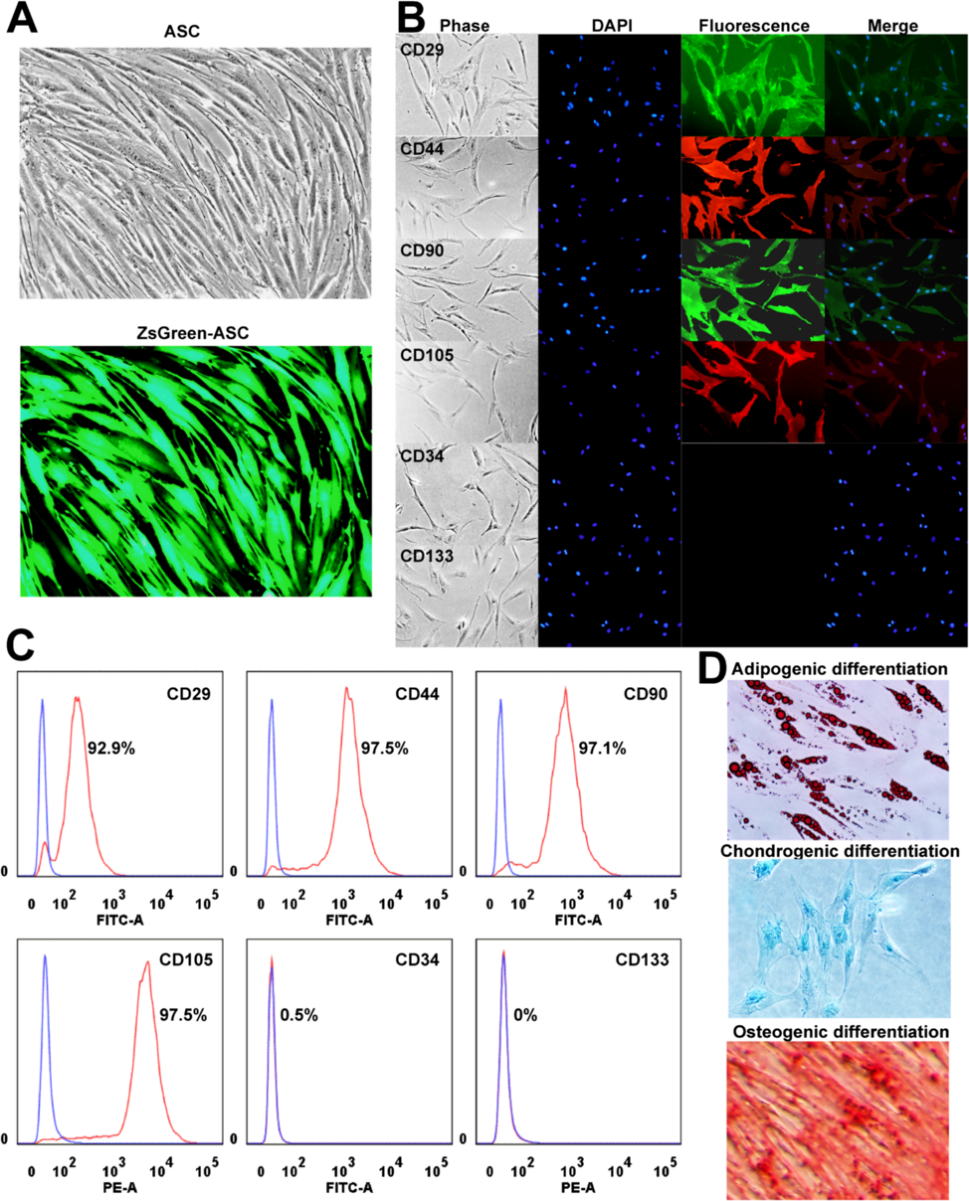
**Characterization of human adipose tissue-derived stem cells (ASCs). (A)** Morphology of ZsGreen-positive ASCs was observed under bright-field and green fluorescence. **(B)** Immunostaining indicated that ASCs were positive for cell surface markers CD29, CD44, CD90, and CD105 and negative for CD34 and CD133. **(C)** Flow cytometry confirmed that the cells were negative for CD34 and CD133 but that CD29, CD44, CD90, and CD105 were highly expressed. **(D)** Triplet differentiation assays revealed that ASCs could differentiate into adipocytes, chondrocytes, and osteocytes. Adipocytes, chondrocytes, and osteocytes were stained by Oil Red O, Alcian blue 8GX, and Alizarin red, respectively. DAPI, 4′,6-diamidino-2-phenylindole.

The morphology of expanded human ASCs appeared to be fibroblast-like and shuttle-shaped. To trace the distribution of transplanted ASCs in the ALF rats, we employed the lentivirus expressing ZsGreen to infect human ASCs. Transplanted ASCs and their corresponding progenies were traced by using a fluorescence microscope at 488 nm, and the labeling efficiency was over 90% (Figure 1A). CD29, CD44, CD90, and CD105 were highly expressed, whereas the cells expressed with minimal levels of CD34 and CD133 (Figure 1B). Furthermore, flow cytometry revealed that over 90% cells expressed CD29 (92.9%), CD44 (97.5%), CD90 (97.1%), and CD105 (97.5%) but that almost all of the cells were negative for CD34 (0.5%) and CD133 (0%) (Figure 1C). To definitely characterize the multipotency of human ASCs, we performed triplet differentiation assay, consisting of adipogenic differentiation, chondrogenic differentiation, and osteogenic differentiation. Triplet differentiation assay indicated that the multipotency of cultured ASCs was well maintained (Figure 1D).

### The survival rate and biochemical indicator analysis after adipose tissue-derived stem cell treatment

**Figure 2:**
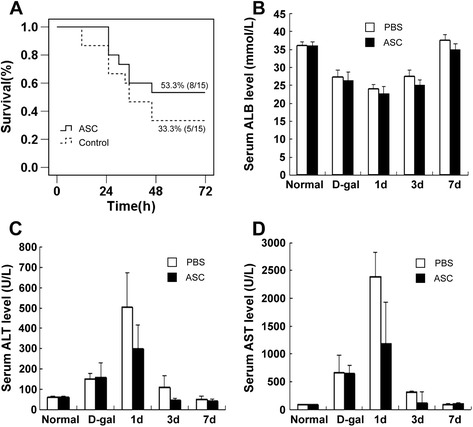
**The survival rate and biochemical indices after adipose tissue-derived stem cell (ASC) treatment. (A)** Survival curves of D-galactosamine (D-gal) induced acute liver failure in the ASC-treated group and the phosphate-buffered saline (PBS)-treated group. The survival rate of the ASC-treated group at each time point was prolonged significantly compared with the PBS-treated group (n = 15, *P* <0.05). **(B)** No significant difference of serum albumin (ALB) levels was observed between the PBS-treated group and the ASC-treated group (n = 5, *P* >0.05). **(C)** The serum alanine aminotransferase (ALT) level of the ASC treatment group (301.7 **±** 116 U/L; 47 **±** 9.1 U/L) was significantly lower than that of the PBS-treated group (506.7 **±** 166 U/L; 112.5 **±** 55.4 U/L) at 1 and 3 days, indicative of liver function improvement (n = 5, *P* <0.01). **(D)** The serum aspartic aminotransferase (AST) level of the ASC-treated group (1,186.7 **±** 426.2 U/L; 128 **±** 19.3 U/L) was significantly lower than that of the PBS-treated group (2,396.7 **±** 743 U/L; 321.3 **±** 185.7 U/L) at 1 and 3 days (n = 5, *P* <0.01).

D-gal is known for inducing the features of acute hepatitis through affecting cell membranes and the synthesis of nucleic acids and proteins and has been used extensively in the development of animal models of ALF [25,26]. Human ASCs were transplanted into spleens of rats 24 hours later after D-Gal injection. All of the animals were followed up for 7 days after transplantation, and the percentage of animals that survived was analyzed. On the first day after transplantation, 100% (15 out of 15) and 86.7% (13 out of 15) of the animals survived in the ASC-treated group and PBS-treated group, respectively. On the second day after treatment, the survival rate of the PBS-treated group decreased to 33.3% (5 out of 15), whereas the survival rate of the ASC-treated group was 53.3% (8 out of 15). After 48 hours, the survival rates of both groups remained the same as the second day after treatment (Figure 2A). In addition, the rats in the PBS-treated group were characterized by aberrant listlessness, lethargy, refusal to eat, and messy hair, whereas the ASC-treated group exhibited a better spirit and the total mortality was 46.7%, suggesting that ASC treatment promoted the viability of ALF rats. We next measured the biochemical indices of both groups to determine whether ASC treatment could improve liver function of ALF rats. Serum ALB levels of both groups decreased obviously within 2 days and returned to the normal level 7 days later after D-gal injection. However, there was no significant difference between the ASC-treated group and the PBS-treated group (Figure 2B). The ALT and AST levels of the PBS-treated group increased to the maximum values after D-gal injection whereas those of the ASC-treated group were obviously lower. Serum ALT and AST levels in both groups were markedly decreased after 3 days of ASC transplantation; however, those of the ASC-treated group were lower than those of the PBS-treated group, indicative of liver function improvement. Serum ALT and AST levels of both groups returned to normal 7 days later (Figure 2C-D).

### Effects of adipose tissue-derived stem cell treatment on cell proliferation and apoptosis

**Figure 3:**
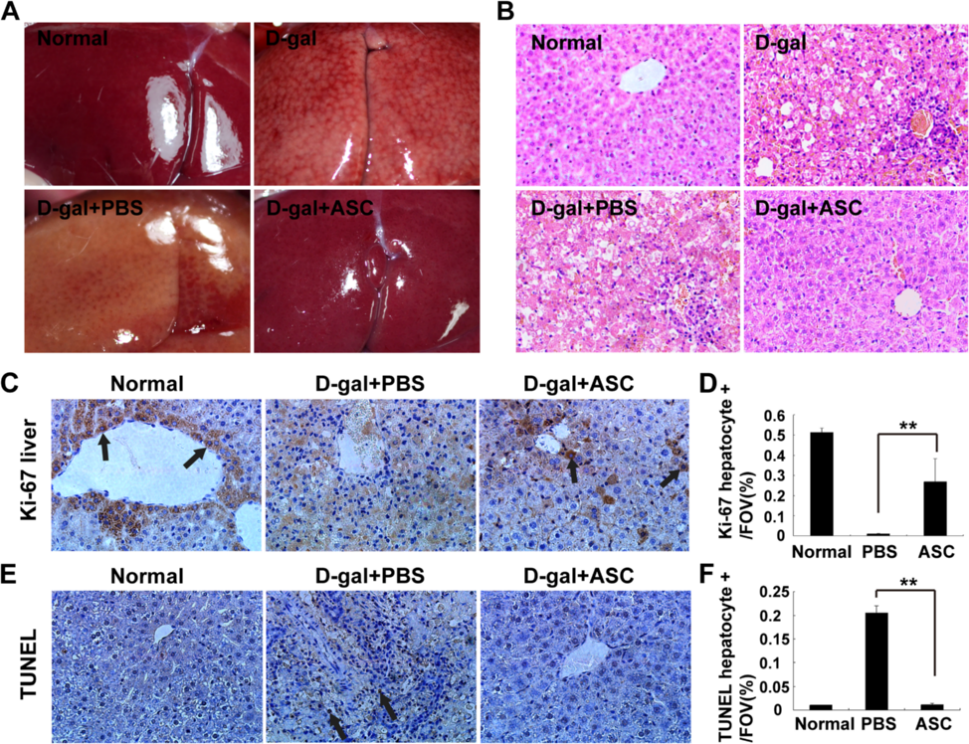
**Liver morphology, cell proliferation, and apoptosis after adipose tissue-derived stem cell (ASC) treatment. (A)** Liver morphology of normal rats, D-galactosamine (D-gal) induced rats, phosphate-buffered saline (PBS)-treated rats, and ASC-treated rats. **(B)** Liver hematoxylin-and-eosin staining sections of normal rats, D-gal induced rats, PBS-treated rats, and ASC-treated rats. The livers of ASC-treated rats showed significant morphologic improvement compared with PBS-treated rats. **(C)** Positive cells were observed by Ki-67 staining (brown) in the liver sections. The ASC-treated group showed more positive cells (black arrows) than the PBS-treated group, suggesting that ASC treatment promoted cell proliferation in liver. **(D)** Quantification analysis of the Ki-67 staining section by Image-Pro Plus software. **(E)** Apoptotic cells (black arrow) were observed in the untreated group by TUNEL (terminal deoxynucleotidyl transferase dUTP nick end labeling) assay. Few apoptotic cells were observed in the ASC-treated group and normal livers, indicating that ASC treatment protected liver cells from apoptosis. **(F)** Quantification of TUNEL-positive cells in the field of view (FOV) by digital image analysis.

The morphology of hepatic tissues was obviously changed after D-gal injection compared with normal liver, given that point or flake congestion was observed on the surface of liver, and the liver was enveloped with a strained and dull capsule. Three days after transplantation, the livers of the PBS-treated group presented ischemic necrosis whereas the ASC-treated group appeared normal (Figure 3A). Correspondingly, HE staining showed that the livers of the PBS-treated group had disorganization of hepatic lobules, hepatic cord disorders, extensive necrosis of hepatocytes, and sinusoidal dilatation. Additionally, the periportal and necrotic areas in the livers of the PBS-treated group contained a plethora of inflammatory cell infiltrations. However, the structure of hepatic tissues in the ASC-treated group appeared normal, suggesting that the liver functional indices of the ASC-treated group were significantly improved (Figure 3B). Given that the proliferation of hepatocytes may contribute to the functional improvement of the injured livers, we next investigated whether the ASC transplantation led to liver cell proliferation by Ki-67 stain. Proliferative cells were distributed particularly around the central vein in the hepatic tissues of the ASC-treated group but not in the PBS-treated group, indicative of the hepatocytic proliferation (Figure 3C). Moreover, quantification analysis revealed that approximately 30% of proliferative cells were increased in the ASC-treated group compared with the PBS group (Figure 3D). To determine whether ASC treatment reduced the number of apoptotic cells, TUNEL assays were used. The liver section of the PBS-treated group was characterized by apoptotic nuclei cells, indicative of hepatocytic apoptosis. In contrast, few apoptotic cells were found in the ASC-treated group, demonstrating that ASC transplantation protected liver cells from apoptosis (Figure 3E). Quantification analysis further revealed that over 20% of apoptotic cells were reduced in the ASC-treated group compared with the PBS-treated group (Figure 3F).

### Cell fate after transplantation

**Figure 4:**
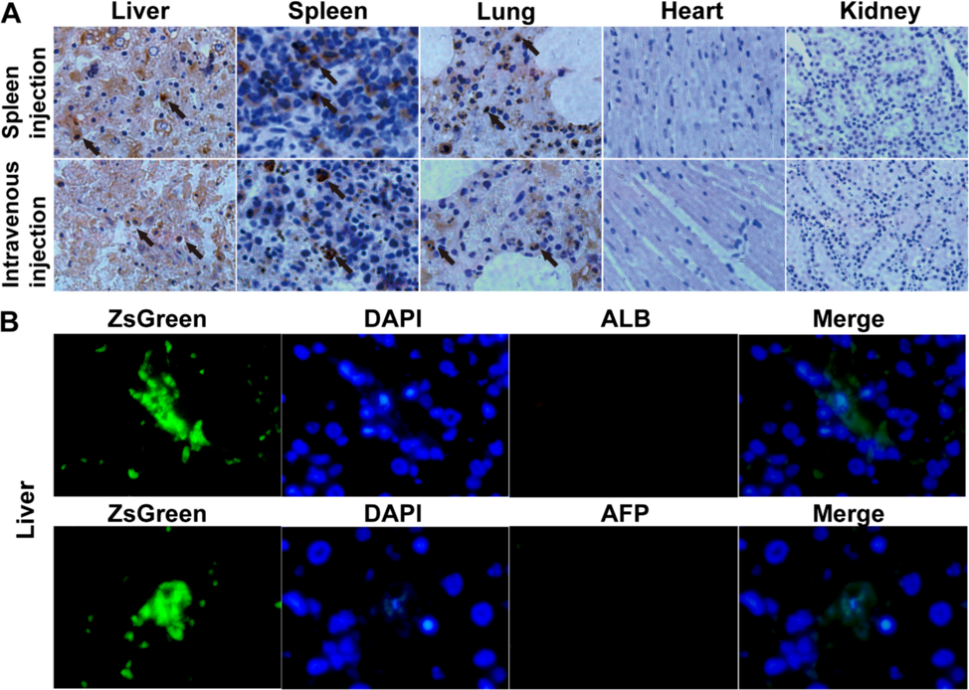
**The cell fate of transplanted adipose tissue-derived stem cells (ASCs) in the liver. (A)** Cell tracing after transplantation. Distribution of transplanted ZsGreen-positive cells were found in the liver, spleen, lung, heart, and kidney after delivery through the spleen or femoral vein. More positive cells (black arrow) were observed in the livers of rats 24 hours after intravenous ASC transplantation through the spleen, indicating that the migration rate to the liver was higher through intravenous transplantation. Moreover, a plethora of transplanted cells persisted in the spleen through spleen delivery. Positive cells were observed in the lung but not in the heart and kidney. **(B)** Double-fluorescence imaging revealed that there was no human albumin (red) or human alpha-fetoprotein (red) expression in the livers, suggesting that transplanted ASCs did not differentiate into hepatocytes. AFP, alpha-fetoprotein; ALB, albumin; DAPI, 4′,6-diamidino-2-phenylindole.

Several delivery methods had been used to transplant cells through peripheral vein, hepatic artery, portal vein, liver, and spleen. To monitor the fate of transplanted cells, we employed the ASCs infected with lentivirus expression ZsGreen to transplant through the spleen and femoral vein. Interestingly, we found that femoral vein delivery showed a higher engraftation rate to the liver than spleen delivery. More ZsGreen-positive cells were observed in the spleen through spleen delivery than intravenous transplantation, suggestive of a plethora of transplanted cells remaining in the spleen. Moreover, a few ASCs migrated to the lung via spleen delivery and showed a similar distribution pattern compared with femoral vein delivery. Of note, there were no transplanted cells engrafting to the heart and kidney in either delivery method. In short, after injection in the spleen and femoral vein, ZsGreen-positive cells were observed in the liver after 24 hours of transplantation, suggesting that transplanted cells could rapidly reach the injury hepatic tissues (Figure 4A). To assess whether the transplanted ASCs differentiated into hepatocytes, we investigated the coexpression of ZsGreen and markers of human hepatocytes, including human ALB and human alpha-fetoprotein. Double-fluorescence immunohistochemistry analysis found no expression of either ALB or alpha-fetoprotein in ZsGreen-positive cells within 3 days after transplantation, indicating that the transplanted ASCs did not differentiate into hepatocytes in the short term (Figure 4B).

### Effects of adipose tissue-derived stem cell (ASC) conditional media and ASC lysate on acute liver failure rats

**Figure 5:**
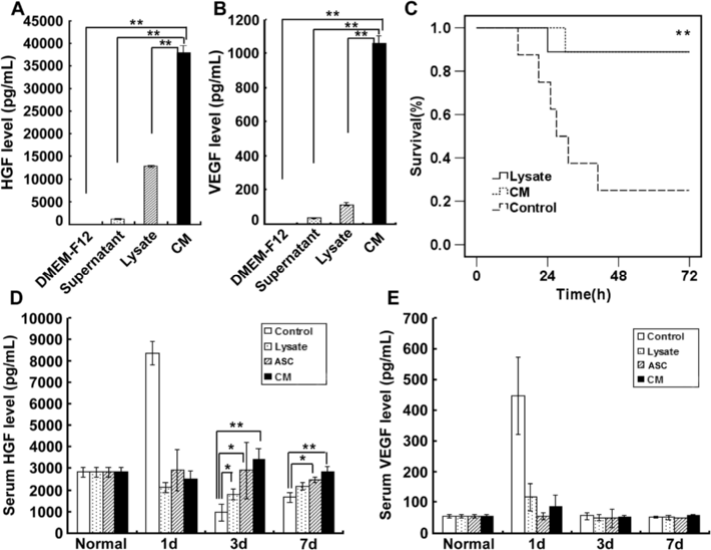
**Levels of key growth factors for liver function. (A)** The hepatocyte growth factor (HGF) levels were determined by enzyme-linked immunosorbent assay (ELISA) in Dulbecco’s modified Eagle’s medium/F12 (DMEM/F12), adipose tissue-derived stem cell (ASC) serum-free culture cell supernatant (supernatant), ASC lysate, and concentrated ASC conditional media (CM) (n = 3). **(B)** The vascular endothelial growth factor (VEGF) levels were determined by ELISA in DMEM/F12, ASC serum-free culture cell supernatant (Supernatant), ASC lysate (Lysate), and concentrated ASC CM (n = 3). **(C)** Survival curve of the DMEM/F12 group (Control), the ASC lysate group (Lysate), and the concentrated ASC CM group. The survival rate of the ASC lysate-treated group and the CM-treated group was significantly higher, implying that factors secreted by ASCs may play key roles in liver failure improvement (phosphate-buffered saline group: n = 8; ASC lysate/CM group: n = 9; *P* <0.01). **(D)** Serum HGF level of the DMEM/F12 group (Control), the ASC lysate group (Lysate), the ASC transplantation group (ASC), and the concentrated ASC CM group (n = 5). **(E)** Serum VEGF level of rats after treatment (n = 5). *P <0.05, **P <0.01.

By ELISA, both HGF and VEGF were accumulated in culture supernatant, cell lysates, and ASC conditional media after 24 hours, suggesting that transplanted ASCs secreted significant amounts of HGF and VEGF (Figure 5A, B). Owing to the high levels of HGF and VEGF secreted by ASCs, we next investigated whether ASC culture supernatant, ASC lysates, or concentrated ASC conditional media had a therapeutic effect on ALF. Interestingly, the survival rate of both the ASC lysate-treated group and the ASC conditional medium-treated group was obviously higher than that of the PBS-treated group (Figure 5C). After 3 days of transplantation, VEGF level did not show any difference between each group whereas the HGF levels in both groups were significantly increased, indicating that both ASC lysate and ASC conditional media markedly upregulated serum HGF level, associating with the restoration of liver function (Figure 5D, E).

## Discussion

Stem cells are characterized by their ability to undergo self-renewal and multilineage differentiation and to form terminally differentiated cells under appropriate conditions [27]. Ideal stem cell types for clinical use should meet the following set of four criteria: (1) efficacy, (2) abundance, (3) minimal invasion, and (4) safety [28]. Although BMSCs are considered a benchmark for clinic purposes, BMSC isolation involves an invasive and painful procedure characterized by low yield [29-31]. On the other hand, ASCs have the potentials to differentiate to chondrocytes, adipocytes, neuron-like cells, and liver-like cells [32,33]. They show strong proliferation ability and present low immunogenicity [34,35]. Furthermore, ASCs appear to be more genetically stable in long-term culture compared with BMSCs [36,37]. Moreover, ASCs can be easily isolated and effectively expanded *in vitro* with intact multipotency under appropriate culture conditions. Recent studies have shown that ASC transplantation displays good therapeutic efficacy on multiple diseases, including liver failure [38]. Herein, our data have demonstrated that human ASC transplantation could efficiently improve the liver function of ALF rats. Although we used human ASCs to transplant into immunocompetent rats instead of immonodeficient recipients, the human ASCs indeed increased the survival rates of the immunocompetent ALF rats. Many other groups had reported that they had successfully transplanted human cells, including human mesenchymal stem cells and human neural stem cells, into immunocompetent rodent hosts and obtained functional outcomes [39-41].

In the present study, although only a few cells migrated to the liver via spleen delivery and most transplanted cells remained in the spleen, ASC treatment indeed attenuated the ALF conditions. Moreover, the therapeutic efficacy was noticeable although the transplanted ASCs did not differentiate into hepatocytes in the injury liver within a short term. Based on these data, we hypothesize that ASC may promote hepatocytic proliferation and inhibit cells apoptosis, resulting in a restoration of liver function by the likelihood of secreting growth factors. Indeed, cytokines secreted by stem cells may play important roles in the regulation of organ function [42,43]. Recent studies have revealed that serum aminotransferase level was significantly decreased by treatment with MSC conditional media, suggesting that factors secreted from stem cells may improve liver function and increase the survival rate of rats with hepatic failure [44]. It has also been documented that the conditional media of liver stem cells distinctly increase the survival rate, promote hepatocytic proliferation, and inhibit hepatocyte apoptosis in treating fulminant hepatic failure in mice [45]. Umbilical cord mesenchymal stem cells have shown a strong ability to promote the regeneration of autologous hepatocytes using a paracrine approach instead of adult hepatocytes [46]. In this study, both ASC lysate and ASC conditional media have demonstrated better therapeutic potential in ALF treatment. Although the VEGF level did not markedly change, the increment of the HGF level was significantly increased in the serum of the ASC-treated group, supporting a view that ASC transplantation may attenuate the ALF conditions by secreting factors valuable to liver function improvement.

In this study, the HGF and VEGF levels were much higher in the serum of the PBS-treated control group than that of the ASC-, lysate-, and conditional media-treated groups at day 1. One possible reason might be that the bodies of the rats have protective effects against the sudden or acute injury through rapidly increasing the levels of growth factors. Particularly, HGF is an important growth factor for the proliferation of hepatocytes. The rapidly increasing HGF levels can compensate the injury of the livers by D-gal. In the ASC, lysate, and conditional media treatment groups, because these factors (ASCs, lysates, and conditional media) might offset the protective effects of the bodies, the levels of HGF and VEGF were much lower than those of the control groups. Other groups had reported similar protective effects in injury. It was reported that HGF levels increased rapidly after ischemia/reperfusion injury [47]. Circulating HGF increased in the early stage of acute myocardial infarction [48]. Moreover, it had been found that HGF was upregulated in the injury liver following CCl_4_ administration [49].

## Conclusions

Our studies have revealed that ASC treatment significantly improves liver function in terms of survival rates in accordance with a better hepatic tissue morphology and biochemical indices. In summary, ASC transplantation may have a better potential for ALF treatment.

## Abbreviations

ALB: albumin
ALF: acute liver failure
ALT: alanine aminotransferase
ASC: adipose tissue-derived stem cell
AST: aspartic aminotransferase
BMSC: bone marrow-derived mesenchymal stem cell
D-gal: D-galactosamine
DMEM/F12: Dulbecco’s modified Eagle’s medium/F12
ELISA: enzyme-linked immunosorbent assay
FBS: fetal bovine serum
HE: hematoxylin and eosin
HGF: hepatocyte growth factor
PBS: phosphate-buffered saline
SD: Sprague Dawley
TUNEL: terminal deoxynucleotidyl transferase dUTP nick end labeling
VEGF: vascular endothelial growth factor
